# Comparison of EM-seq and PBAT methylome library methods for low-input DNA

**DOI:** 10.1080/15592294.2021.1997406

**Published:** 2021-11-17

**Authors:** Yanan Han, Galina Yurevna Zheleznyakova, Yanara Marincevic-Zuniga, Majid Pahlevan Kakhki, Amanda Raine, Maria Needhamsen, Maja Jagodic

**Affiliations:** a Department of Clinical Neuroscience, Karolinska Institutet, Center for Molecular Medicine, Karolinska University Hospital, Stockholm, Sweden; b Department of Medical Sciences, Science for Life Laboratory, Uppsala University, Uppsala, Sweden

**Keywords:** Low input DNA, methylome, EM-seq, PBAT

## Abstract

DNA methylation is the most studied epigenetic mark involved in regulation of gene expression. For low input samples, a limited number of methods for quantifying DNA methylation genome-wide has been evaluated. Here, we compared a series of input DNA amounts (1–10ng) from two methylome library preparation protocols, enzymatic methyl-seq (EM-seq) and post-bisulfite adaptor tagging (PBAT) adapted from single-cell PBAT. EM-seq takes advantage of enzymatic activity while PBAT relies on conventional bisulfite conversion for detection of DNA methylation. We found that both methods accurately quantified DNA methylation genome-wide. They produced expected distribution patterns around genomic features, high C-T transition efficiency at non-CpG sites and high correlation between input amounts. However, EM-seq performed better in regard to library and sequencing quality, i.e. EM-seq produced larger insert sizes, higher alignment rates and higher library complexity with lower duplication rate compared to PBAT. Moreover, EM-seq demonstrated higher CpG coverage, better CpG site overlap and higher consistency between input series. In summary, our data suggests that EM-seq overall performed better than PBAT in whole-genome methylation quantification of low input samples.

## Introduction

DNA methylation is a well-studied epigenetic mark, where a methyl-group is covalently bound, most commonly at the 5th carbon of cytosine within CpG dinucleotides. By regulating gene expression, genomic imprinting, X-chromosome inactivation and transposon repression, 5-methylcytosine (5mC) participates in essential developmental processes, and abnormal methylation states can lead to various diseases [1–3].

Several methods have been developed to identify and quantify DNA methylation across the genome. Among them, whole-genome bisulfite sequencing (WGBS) uses bisulfite conversion to detect methylation at single-base resolution. Unmethylated cytosines are converted to uracils after treatment with sodium bisulfite, while 5mC and 5-hydroxymethylcytosine (5hmC) are protected and remain unchanged. Although widely used, a major disadvantage of bisulfite conversion is substantial DNA degradation, which compromises input DNA quality and can introduce bias [4]. Different library strategies are available for whole-genome single-base 5mC quantification but only few can be utilized on samples with low DNA input. DNA amounts obtainable from tissues and cells can be quite low, and at the same time those cells may play a vital role in developmental processes or disease pathogenesis. Post-bisulfite adaptor tagging (PBAT) has been optimized to identify genome-wide methylation even at the single-cell level [5]. But this method, as other commonly used protocols, often leads to high duplication rate with low library complexity, a common disadvantage of BS-based methods. Another method suitable for low-input DNA amounts is enzymatic methyl-seq (EM-seq) [6], a newly developed enzyme-based method. The method uses ten-eleven translocation dioxygenase 2 (TET2) to convert 5mC into 5hmC, 5-formylcytosine and 5-carboxycytosine (5caC) in three consecutive processes. Simultaneously, T4 phage b-glucosyltranferase is applied to convert 5hmC to 5-(β-glucosyloxymethyl) cytosine (5gmC). In the final step, apolipoprotein B mRNA editing enzyme, catalytic polypeptide-like 3A, converts unmethylated cytosines into uracils, while 5caC and 5gmC remain unchanged. Consequently, methylated and unmethylated cytosines can be distinguished by subsequent sequencing. This enzyme-based method circumvents bisulfite-induced damage to the DNA, consequently reducing sample loss [7].

Here, we evaluated two different whole-genome methylome library strategies designed for low-input amounts, single-cell adapted PBAT [5] and EM-seq protocols [6]. Libraries were constructed from series of diluted input DNA amounts (1, 2, 5 and 10ng) derived from two independent samples, which were subsequently sequenced, evaluated and compared.

## Materials and methods

### Low-input DNA samples

Lumbar puncture was conducted in 2015/16 to collect cerebrospinal fluid (CSF) from two females diagnosed with inflammatory disease, aged 60 and 29, respectively. CSF samples, which contained approximately 2.96 and 1.30 million cells, respectively, were collected in 2x15ml size Falcon tubes and centrifuged immediately at 440 g for 10 minutes at RT to separate cells and larger particles from CSF supernatants. CSF cells were subsequently pooled, concentrated to a volume of 20–60 µl and transferred to a 2 ml polypropylene tube, snap-frozen on dry-ice and stored at −80°C until further use. DNA was isolated using QIAmp DNA Mini kit (Qiagen) according to the manufacture’s protocol and DNA concentrations were measured using the DNA high sensitivity Qubit assay (Thermo Fisher Scientific). Each sample was divided in two for EM-seq and PBAT library methods, respectively. In total, 16 whole-genome methylome libraries were generated. In EM-seq libraries, prior to library preparation, the DNA was sheared (sonicated) with Covaris E220 aiming for an average insert size of ~350 bp. And for PBAT libraries, the DNA was fragmented in the BS-conversion step. The study was approved by the Regional Ethical Board (2009/2107-31/2) and all participants signed the informed consent.

### EM-seq library preparation

Libraries were prepared with 1, 2, 5 or 10ng DNA using the NEBNext® Enzymatic Methyl-seq (EM-seq™) kit and Unique Dual Index Primer pairs (New England Biolabs) input according to manufacturer’s instructions (NEB #E7120 S/L v6.0_3/2). Unmethylated lambda phage DNA and plasmid pUC19 DNA, methylated at CpG sites, were added as negative and positive controls, respectively. A total of 12 amplification cycles were used for 1ng of input DNA and 10 cycles for other input amounts. Libraries were pooled equimolarly and then sent for sequencing.

### PBAT library preparation

1, 2, 5 or 10ng of DNA was bisulfite converted using the Imprint DNA Modification Kit (Sigma) followed by incubation at 99°C for 6 min, 65°C for 80 min, 95°C for 3 min and 65°C for 20 min and purified according to the manufacturer’s protocol. DNA was eluted in 10 mM Tris-Cl (pH 8.5) and mixed with 0.4 mM dNTPs, 0.4 µM oligo 1 (Biotin) (CTACACGACGCTCTTCCGATCTNNNNNNNNN) and 1X NEBuffer to a final reaction volume of 24 µl. Samples were incubated at 65°C for 3 min followed by 4°C pause, 5 U of Klenow exo – (New England Biolabs) was added and the incubation was continued at 4°C for 5 min, +1°C/15s to 37°C, 37°C for 90 min. Samples were then incubated with 20 U exonuclease I (New England Biolabs) for 1 h at 37°C. DNA was purified with 0.8X Agencourt Ampure XP beads (Beckman Coulter), eluted in 10 mM Tris-Cl (pH 8.5) and incubated with washed M-280 Streptavidin Dynabeads (Invitrogen) for 30 min with rotation at RT. Beads were washed twice with 0.1 N NaOH, twice with 10 mM Tris-Cl (pH 8.5) and resuspended in 47 µl of 0.4 mM dNTPs, 0.4 µM oligo 2 (TGCTGAACCGCTCTTCCGATCTNNNNNNNNN) and 1X NEBuffer 2. Samples were then incubated at 95°C for 45s followed by 4°C pause before addition of 10 U Klenow exo – and then incubated at +1°C/15s to 37°C, 37°C for 90 min. Beads were washed with 10 mM Tris-Cl (pH 8.5) and resuspended in 50 µl of 0.4 mM dNTPs, 0.4 µM PE1.0 forward primer (AATGATACGGCGACCACCGAGATCTACACTCTTTCCCTACACGACGCTCTTCCGATCT), 0.4 µM indexed iPCRTag reverse primer, 1 U KAPA HiFi HotStart DNA polymerase (Roche) in 1X HiFi Fidelity Buffer. Libraries were amplified by PCR as follows: 95°C 2 min, 10–12 cycles of 98°C 80s, 65°C 30s, 72°C 30s, followed by 72°C 3 min and 4°C hold. 12 amplification cycles were used for 1ng of input DNA and 10 cycles for other input amounts. Amplified libraries were purified using 0.8X Agencourt Ampure XP beads. Quality and quantity of each library was determined using High-Sensitivity DNA chips on the Agilent Bioanalyzer, and the KAPA Library Quantification Kit (Roche). Libraries were pooled equimolarly and then sent for sequencing.

### Sequencing

All libraries of each type were pooled and sequenced 2 × 150 bp paired end on one SP flow cell, NovaSeq 6000 system (v1.0). 10% spike-in phage PhiX was included both in EM-seq and PBAT sequencing runs.

### Data analysis

The Nf-core/methylseq bioinformatics pipeline was applied to fastq sequencing files of all 16 samples with default parameters[8,9]. Taken into consideration that EM-seq generates longer fragments than conventional WGBS libraries, the beta version of nf-core/methylseq, was used with --em_seq parameter. The normal version 1.5 was applied to PBAT with --pbat --clip_r1 9 --clip_r2 9 --three_prime_clip_r1 9 --three_prime_clip_r2 9. Versions and references of tools called by Nf-core/methylseq have been listed in Table S1. Briefly, raw reads were trimmed with Trim Galore to remove adapters and low-quality reads. Trimmed reads were then mapped to the GRCh38 reference genome by Bismark [10] and duplicated reads were removed. Methylation sites were then extracted by Bismark. Upon CpG site identification, coverage and DNA methylation levels were retrieved. Reads mapped to both strands were merged to the coverage of specific CpGs. Methylation levels were calculated by dividing the number of methylated reads by the sum of methylated and unmethylated reads. During data processing, multiple quality control tools were applied: FastQC to identify reads quality, Qualimap [11] to report the alignment quality, Preseq to examine sample complexity and multiQC [12] was applied for the integrated quality control. Genomic features files were downloaded from Ensembl [13].

Plots were generated in local R primarily with package ggplot2. R Package eulerr was used to produce Venn diagrams. Ggpubr was used to arrange sub graphs into one main figure. UpSetR was used to illustrate intersecting sites between libraries in Fig. S3. The landscape plots were produced by CpGtools [14]. All software and R package versions were listed in Table S1.

## Results

### Characteristics of EM-seq and PBAT libraries

**Figure 1.**
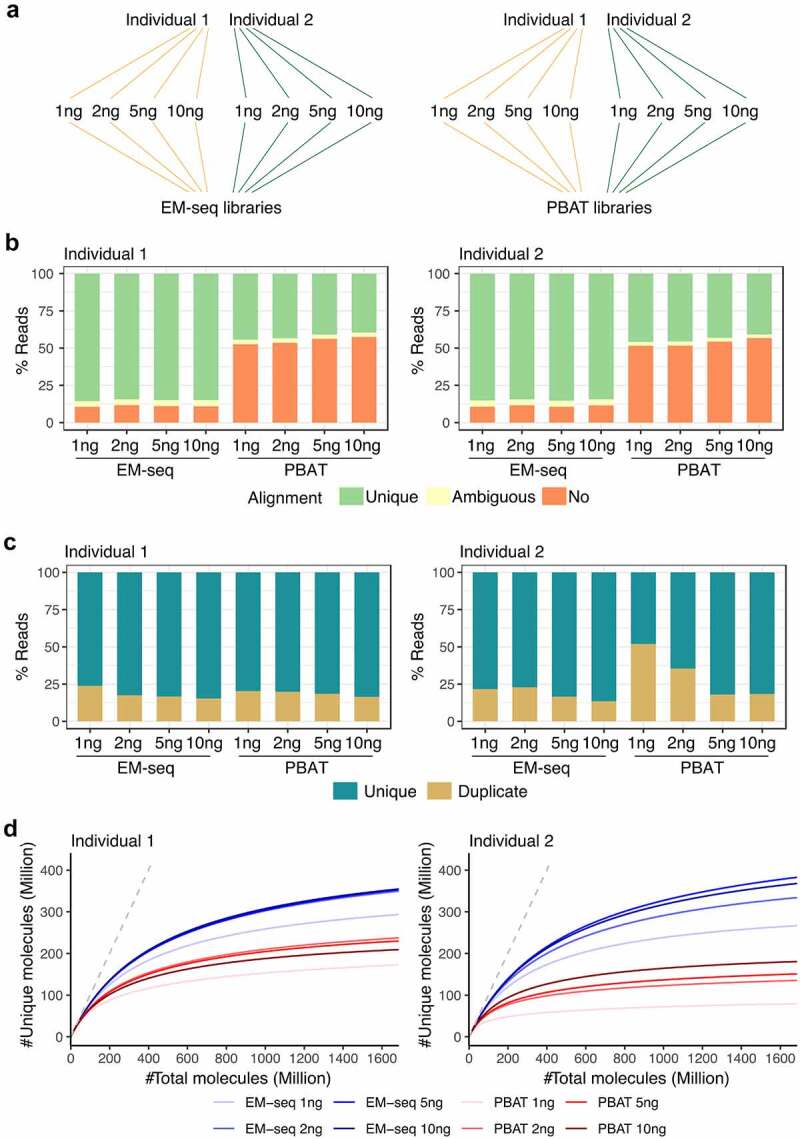
EM-seq libraries performed better in regards to library and sequencing quality. (a) Samples from two individuals (yellow and green) were collected and split into two for EM-seq and PBAT libraries, respectively. Each method included 1, 2, 5 and 10ng input amounts. (b) Stacked bar plots illustrate unique (green), ambiguous (yellow) and no (orange) alignment based on % reads. (c) Illustration of unique (dark turquoise) and duplicate (yellow) reads. (d) Complexity curves of EM-seq (blue) and PBAT (red) library methods. The dashed grey line illustrates maximum library complexity.

A series of low input amounts, 1, 2, 5 and 10ng, derived from samples of two individuals were evaluated with EM-seq and PBAT, which was adapted from the single-cell method [5,6] (Figure 1(a)). The diagrams of these two methods are illustrated in Fig. S1. Library details, including number of PCR cycles, library concentrations and fragment sizes, are provided in Table S2. We calculated methylation level of detected cytosines in CHG and CHH context to approximate the conversion efficiency. Conversion rates of all libraries exceeded 97.1% (mean: 99.2%, median: 99.0%) (Table S3). Concordantly, the calculated methylation levels of unmethylated lambda control DNA in EM-seq were low (less than 0.22% in all samples except individual 1 1ng with 2.88% methylation level) while the pUC CpGs were hyper-methylated (≥94.87%) as expected (Table S3). The numbers of raw, clean, aligned, duplicate and unique reads for all 16 libraries are provided in Table S4.

Furthermore, all 16 libraries showed high sequencing quality with average Phred scores higher than 30 (Fig. S2A). They performed similarly in regards to the R1 strand quality, while for the R2 strand, EM-seq consistently showed higher Phred scores than all four PBAT libraries in both individuals (Fig. S2A). EM-seq produced longer fragments as illustrated by the insert size distribution, where both individuals displayed the majority of inserts >300bp, compared to PBAT libraries, where the majority of inserts were <300bp (Fig. S2B). The mean insert size of EM-seq libraries peaked at 349bp while the mean insert size for PBAT peaked at 148bp (Fig. S2B). In addition, EM-seq demonstrated higher unique alignment rate compared to PBAT libraries in both individuals and across all four input amounts with the average unique alignment of 84.9% and 43.0%, respectively (Figure 1(b), Fig. S2C). The majority of libraries displayed duplication rates below 25%, except 1ng and 2ng PBAT libraries for individual 2, where 52% and 35% of the reads were duplicates and consequently removed (Figure 1(c), Fig. S2D). This observation suggests a duplication discordance for low-input PBAT libraries, however, technical replicates, which were not conducted in this study, would be needed to confirm this. Moreover, the EM-seq libraries displayed higher complexity across all four input amounts (Figure 1(d)). From this evaluation, libraries built by the EM-seq protocol had longer fragments, were more diverse and more consistent across input amounts from both individuals.

### EM-seq covered more CpG sites

**Figure 2.**
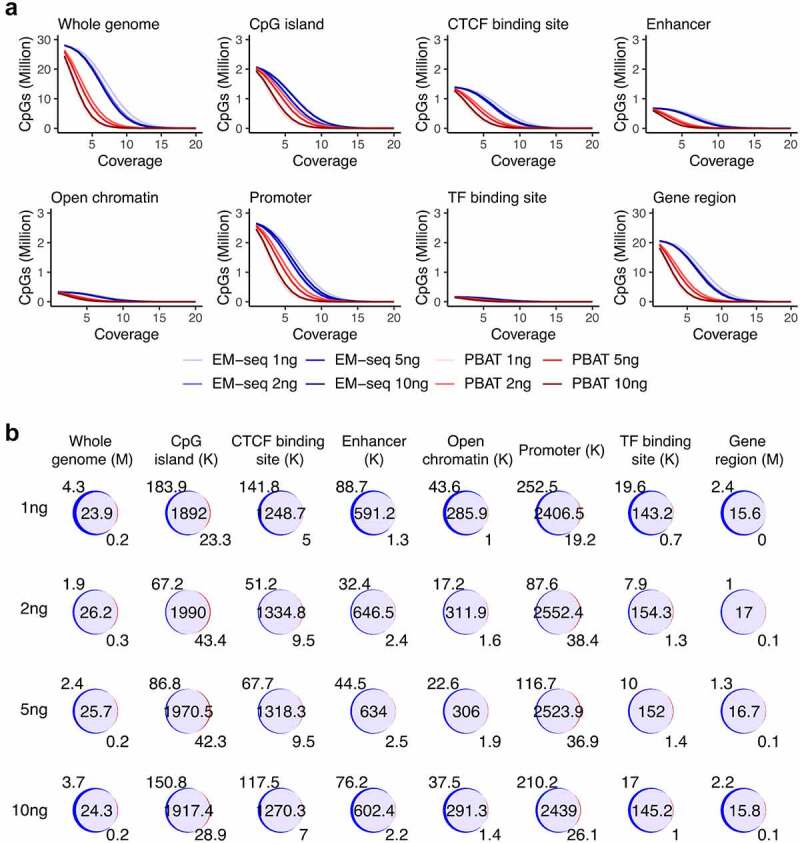
CpG coverage and overlap. (a) Number of CpG sites and sequencing coverage in EM-seq (blue) and PBAT (red) for all input amounts for individual 1 encompassing whole genome and genomic features such as CpG islands, CTCF binding sites, enhancers, open chromatin, promoters, transcript factor (TF) binding sites and gene regions. (b) Overlap (purple) of EM-seq (blue) and PBAT (red) CpG sites from input amounts of 1, 2, 5 and 10ng in individual 1. M (million) for whole genome and gene region, K (thousand) for other features.

We then investigated which method produced overall higher coverage. The majority of CpG sites were covered by the two methods. In total, 28.0, 28.2, 28.1 and 28.0 million CpGs on average were covered in EM-seq inputs of 1, 2, 5 and 10ng, respectively, while 24.0, 26.3, 24.2 and 24.7 million CpGs were covered in PBAT libraries. All input EM-seq libraries yielded higher coverage than the four PBAT libraries in both individuals in particularly at low-end coverage cut-offs (Figure 2(a), Fig. S4A). At 5X coverage cut-off, 20.1, 22.5, 21.1 and 18.8 million CpGs were covered by EM-seq libraries, while only 3.2, 8.5, 4.4 and 4.1 million CpGs were covered by PBAT libraries calculated as the mean of two individuals in the four input amounts. For the 10X cut-off, 4.0, 6.5, 4.4 and 2.7 million CpGs were covered in EM-seq while 76.7, 513.2, 177.6 and 100.4 thousand CpGs in PBAT libraries. Noticeably, CpG sites showed high overlap among different inputs in both individuals (Figure 2(b), Fig. S4B). The mean percentage of PBAT CpGs that were also detected with EM-seq was 99.1%, 99.3%, 99.2% and 99.0% corresponding to 1, 2, 5 and 10ng, respectively. Furthermore, CpG sites overlapping in all four libraries were the main group compared to other intersection groups both for the EM-seq and PBAT libraries in the two individuals (Fig. S3). The coverage of EM-seq libraries was higher than PBAT libraries not only overall in the whole genome, but also across various genomic features including CpG islands, CTCF binding site regions, enhancers, open chromatin, promoters, transcription factor (TF) binding regions and gene regions from transcriptional start sites (TSS) to transcriptional end sites (TES) (Figure 2(a), Fig. S4A). Furthermore, overlap analysis revealed a large fraction of common CpG sites between the two methods (Figure 2(b), Fig. S4B). Moreover, IGV genome browser snapshots of the L1MB4, L1M4 and L1PA3 region, which is classified as a LINE-1 retrotransposable element, revealed that EM-seq displayed higher coverage compared to PBAT in this challenging region (Fig. S5). Taken together, EM-seq covered more CpG sites and contained the majority of CpGs detected by PBAT.

### EM-seq and PBAT generated expected DNA methylation patterns

**Figure 3.**
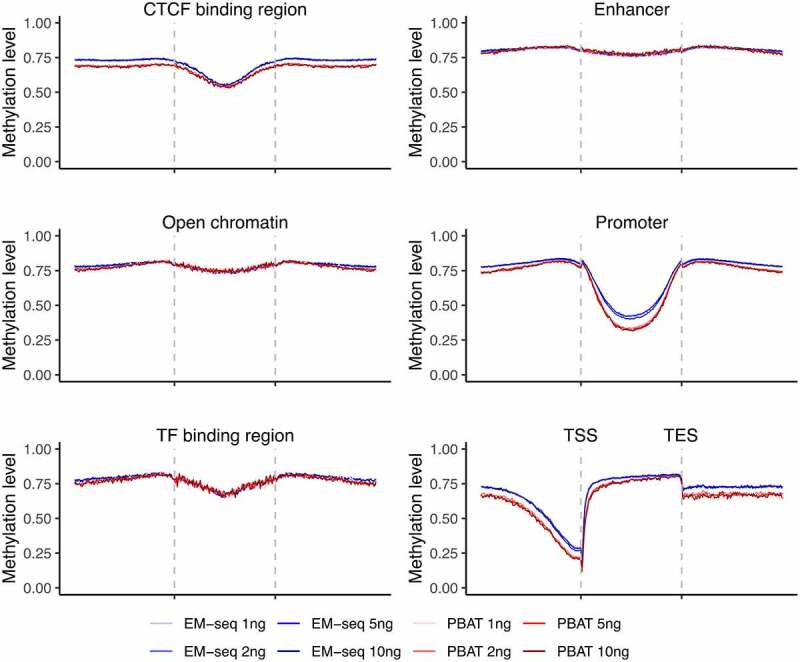
Landscapes of DNA methylation levels within genomic features. Illustration of methylation levels around CTCF binding site regions, enhancers, open chromatin, promoters, TF binding regions, and gene region from TSS to TES. EM-seq libraries are illustrated in blue and PBAT in red for different input amounts (1, 2, 5 and 10ng) in individual 1. CpGs with at least 5X were considered.

Next, we wanted to know if the two methods captured expected DNA methylation patterns across different genomic features. We applied CpGtools to analyse the methylation landscape for all 16 libraries. Distinct genomic features displayed different DNA methylation patterns in a similar manner between individuals (Figure 3, Fig. S6C). Within gene regions, introns displayed higher DNA methylation levels, especially among internal introns and 5' UTR regions (Fig. S6A, Fig. S6B). Other genomic features, such as open chromatin and enhancers, also displayed characteristic patterns with overall higher DNA methylation levels, except for central CTCF binding regions, promoter regions, and upstream TSS which were as expected lower (Figure 3, Fig. S6C). This general pattern was observed in all libraries. In some genomic features, like enhancers and TF binding regions, the two methods performed similarly (Figure 3, Fig. S6C). However, for some regions, different features performed slightly different between EM-seq and PBAT libraries (Figure 3, Fig. S6C). For example, the four EM-seq libraries showed somewhat higher DNA methylation levels compared to the four PBAT libraries in upstream gene regions and upstream CTCF binding regions (Figure 3, Fig. S6C). But these differences were limited and not observed in other features and coverages. In summary, expected DNA methylation distribution was demonstrated in all libraries, which suggests that reliable DNA methylation patterns were detected with both methods and all four input amounts.

### EM-seq showed higher correlation of DNA methylation levels

**Figure 4.**
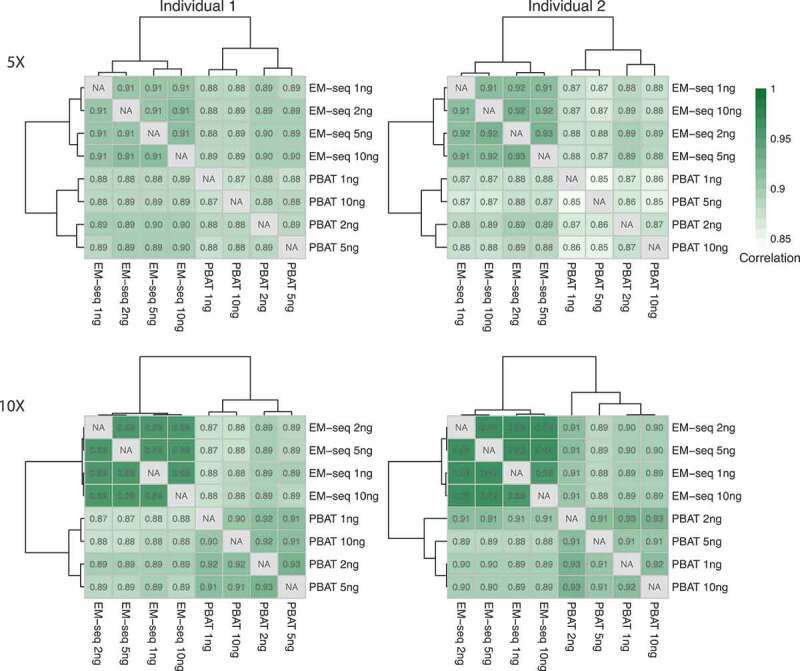
Correlation of DNA methylation levels. Heatmaps illustrating Pearson’s correlation coefficients between input amounts (1, 2, 5 and 10ng) and library methods (EM-seq and PBAT). CpGs covered by at least 5X and 10X were considered.

Our final evaluation was the comparison of DNA methylation levels between samples. We calculated Pearson’s and Spearman’s rank correlation coefficients between the two library protocols, EM-seq and PBAT, and the four input amounts, 1, 2, 5 and 10ng, respectively (Figure 4, Fig. S7). CpG sites with at least 5X coverage were considered, for which DNA methylation distribution was demonstrated in Fig. S8. All EM-seq libraries displayed Pearson’s correlation coefficients ≥0.91 and PBAT libraries ≥0.85, while Spearman’s rank correlation coefficients were ≥0.82 for EM-seq and ≥0.74 for PBAT libraries, respectively. Of note, even the 1ng library displayed good correlation with ≥0.91 for EM-seq and ≥0.85 for PBAT Pearson’s and ≥0.82 EM-seq and ≥0.74 PBAT Spearman’s rank correlation coefficients, respectively (Figure 4, Fig. S7). Application of the 10X coverage filter revealed concordant Pearson’s and Spearman’s correlation coefficients (Figure 4, Fig. S7). Furthermore, EM-seq also presented more bimodal distribution of DNA methylation levels than PBAT (Fig. S8). Overall, both methods performed well with low input amounts, when considering Pearson’s correlation coefficients, and again the EM-seq method displayed a measurable better performance over PBAT.

## Discussion

We compared two methods that are suitable for low input amounts, namely EM-seq [6] and single-cell adapted PBAT [5]. Both methods estimated DNA methylation genome-wide, produced expected DNA methylation landscapes around genomic features and displayed high level of correlation across input amounts. However, we found that EM-seq performed quantitatively better in all comparisons. Firstly, EM-seq libraries showed longer insert sizes, higher alignment rates and lower duplication rates. Secondly, EM-seq covered more CpG sites and encompassed nearly all CpG sites detected by PBAT. Thirdly, EM-seq illustrated higher DNA methylation level correlation between libraries across all DNA input amounts (1–10ng).

Accuracy of estimated DNA methylation levels is influenced by the sequencing coverage as previously demonstrated for PBAT [15,16]. EM-seq encounters the same situation due to the same calculation principle. PCR amplification and conversion efficiency can also bring bias [16]. In our study, EM-seq generally displayed a higher level of library complexity (Figure 1(d)), which upon additional sequencing, could have increased the coverage. On the other hand, low complexity libraries are not expected to gain much in regards to coverage with additional sequencing, due to a likely, concordant increase in read duplication, which would consequently be filtered during pre-processing. Hence, better precision and capturing of additional CpGs are expected with higher complexity libraries. The slight discrepancy we detect in DNA methylation levels between EM-seq and PBAT in some genomic features could indeed be due to a compromised coverage. To gain an adequate number of sites for comparison, we included a 5X threshold, which is in the low-end for DNA methylation estimation. However, the discrepancy was small and overall DNA methylation landscapes for both methods and all input amounts performed as expected (Figure 3). Furthermore, we tested that the higher DNA methylation levels detected for EM-seq were not due to an insufficient enzymatic reaction, which could potentially fail to properly distinguish methylated and unmethylated sites. For this, we tested percentages of methylated cytosines in non-CpG (i.e., CHG and CHH) context and detected an efficiency above 97.1% in all libraries (Table S3). For PBAT libraries, we detected a comparable conversion efficiency (Table S3). Hence, we could conclude that in our EM-seq and PBAT libraries non-methylated cytosines were sufficiently converted for all input amounts, which is crucial for accurate estimation of DNA methylation levels.

Bisulfite treatment is relatively harsh on genomic DNA and can cause widespread degradation. A major difference between EM-seq and PBAT, of which the latter is based on bisulfite-conversion, was indeed fragmentation as illustrated when plotting the insert size (Supp. Figure 1(b)). Paired-end sequencing with read lengths of 150nt should optimally present the majority of insert sizes > 300bp, which was indeed the case for EM-seq. However, PBAT library insert sizes peaked <300bp, suggesting that sequencing conditions were not optimal. Due to lower complexity of *in silico* converted reference genomes used for mapping of EM-seq and PBAT data, paired-end and longer sequencing reads are an advantage to avoid ambiguous mapping [17]. Hence, longer insert sizes, and thereby less degraded genomic DNA, as demonstrated by EM-seq, is preferential.

To estimate conversion rates for EM-seq libraries, unmethylated lambda phage DNA and CpG methylated pUC19 vector DNA, were added as controls. Furthermore, we estimated conversion rates based on CHG and CHH methylation for both EM-seq and PBAT libraries. Lambda phage, CHG and CHH methylation were all <3%, thus, the conversion efficiency was >97%. Noticeably, lambda phage and non-CpG methylation were all highly compatible. Although the majority of EM-seq samples showed higher conversion efficiency compared to PBAT, the 1ng sample in individual 1 had lower efficiency. Noticeable, EM-seq 1ng individual 2 had good conversion efficiency, hence, the EM-seq method could potentially be more fluctuant in low-input samples.

EM-seq is a comparatively new method but has been successfully used in other studies too and previously shown to perform well by its higher mapping efficiency [6,18–21]. In a study on Arabidopsis thaliana, EM-seq was for example recommended over WGBS due to its higher mapping rate and coverage, lower duplication rate, lower influence by experimental condition, and higher consistency between replicates [21]. Here, we also found that EM-seq performed better than PBAT for low input amounts.

Although PBAT overall did not perform as well as EM-seq in our study, the methodology may be better adapted for specific applications such as quantification of DNA methylation on the single-cell level [5], potentially for some specific cell types etc. The lowest reported input for EM-seq libraries on the other hand is 100pg, which suffered from high duplicate rate [5,6]. Since EM-seq is a relatively new method, further exploration and optimization may bring the input down. Here we show that for the input ranges between 1–10ng, roughly corresponding to 200 to 2000 cells, EM-seq outperformed PBAT. Hence, the balance between advantages and drawbacks between different methods should be thoroughly considered based on sample situation and study aim.

An advantage of EM-seq is the enzymatic deamination step, which is more gentle than bisulfite and thereby causes less damage to the DNA compared to PBAT, which relies on the much harsher bisulfite treatment for C > T conversion. However, an advantage of the PBAT protocol is the post-bisulfite adapter tagging, which circumvents bisulfit-induced degradation of sequencing templates. In the future, it would be interesting to see the two methods combined, i.e., enzymatic deamination followed by adapter tagging, which would protect the DNA and potentially generate more complex sequencing libraries that are crucial for low-input samples.

## Supplementary Material

Supplemental Material
