# Supplementary material for: Comparison of EM-seq and PBAT methylome library methods for low-input DNA
Source: Epigenetics. 2021 Nov 17;17(10):1195–204. doi: 10.1080/15592294.2021.1997406 (PMC9542412; doi:10.1080/15592294.2021.1997406)
Supplement: Supplemental Material [file KEPI_A_1997406_SM2660.docx]

**Supplementary figures**
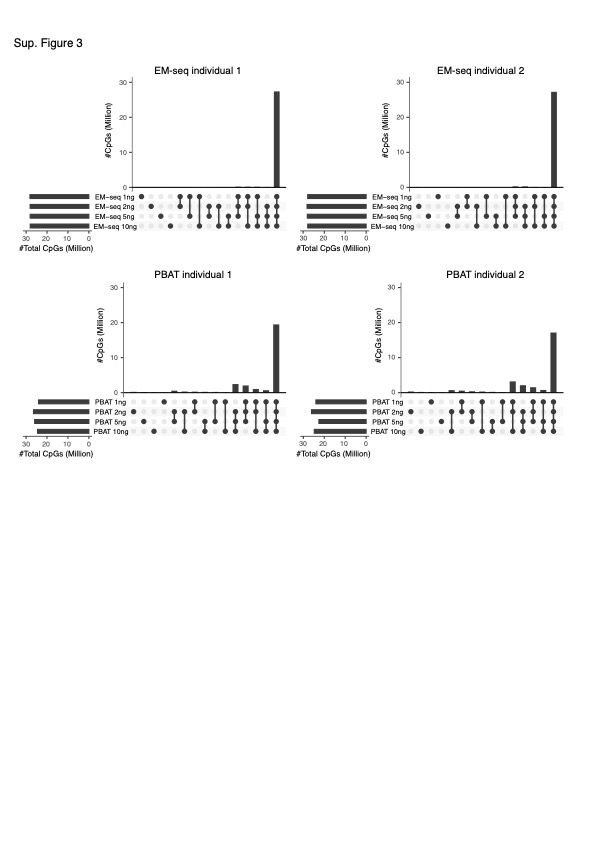

**Figure Captions**

Figure S1. Diagram of EM-seq and PBAT schemas.

Figure S2. EM-seq libraries performed better in multiple quality control steps. (A) Illustration of Phred scores for R1 and R2 strand reads in EM-seq (blue) and PBAT (red) libraries for all input amounts (1, 2, 5 and 10ng). (B) Illustration of insert size, from which fragment size and input DNA quality can be inferred. (C) Stacked bar plots illustrating unique (green), ambiguous (yellow) and no (orange) alignment based on number of reads. (D) Number of unique (dark turquoise) and duplicate (orange) reads.

Figure S3. Covered CpG sites in intersection groups between 1, 2, 5 and 10ng in EM-seq libraries and PBAT libraries for the two individuals. Total number of CpGs in each library were bar plotted in the left bottom panel and the CpG number in each intersect group were in the right panel. Most CpGs overlapped in all 1, 2, 5 and 10ng input libraries for both library methods and EM-seq libraries covered more aggregated and overlapped CpG sites.

Figure S4. CpG coverage and overlap in individual 2 (A) Number of CpG sites and sequencing coverage in EM-seq (blue) and PBAT (red) for all input amounts for individual 2 encompassing whole genome and genomic features such as CpG islands, CTCF binding sites, enhancers, open chromatins, promoters, TF binding sites and gene regions. (B) Overlap (purple) of EM-seq (blue) and PBAT (red) covered CpG sites (million for whole genome and gene region, thousand for the other features) from input amounts of 1, 2, 5 and 10ng in individual 2 for whole genome region and genomic feature region. M (million) for whole genome and gene region, K (thousand) for other features.

Figure S5. Read coverage of L1MB4, L1M4 and L1PA3 region.

Figure S6. Methylation level distribution within and around gene regions in (A) individual 1, (B) individual 2 and (C) genomic features for individual 2. Sites with more than 5X coverage were included.

Figure S7. Spearman’s rank correlation of DNA methylation levels. Heatmaps illustrate Spearman’s rank correlation coefficients between input amounts (1, 2, 5 and 10ng) and library methods (EM-seq and PBAT). CpGs covered by at least 5X and 10X were considered.

Figure S8. Density distribution of DNA methylation levels. Distribution of DNA methylation levels for EM-seq (blue) and PBAT (red) library methods in two individuals. CpG sites with at least 5X or 10X coverage were considered.

**Supplementary Tables**

Table S1. Software and packages

| **Software & package** | **Version** | **Reference** |
| --- | --- | --- |
| Nextflow | 20.01.0 | [8] |
| nf-core/methylseq | 1.5 | [9] |
| FastQC | 0.11.9 | <https://www.bioinformatics.babraham.ac.uk/projects/fastqc/> |
| Cutadapt | 2.9 | <https://cutadapt.readthedocs.io/en/stable/index.html> |
| Trim Galore! | 0.6.4_dev | <https://www.bioinformatics.babraham.ac.uk/projects/trim_galore/> |
| Bismark | 0.22.3 | [10] |
| Qualimap | 2.2.2-dev | [11] |
| Preseq | 2.0.3 | <http://smithlabresearch.org/software/preseq/> |
| MultiQC | 1.8 | [12] |
| R | 4.0.2 | https://www.R-project.org/ |
| ggplot2 | 3.3.3 | https://ggplot2.tidyverse.org |
| eulerr | 6.1.0 | https://cran.r-project.org/package=eulerr |
| ggpubr | 0.4.0 | https://CRAN.R-project.org/package=ggpubr |
| UpSetR | 1.4.0 | https://CRAN.R-project.org/package=UpSetR |
| CpGtools | 1.0.6 | [14] |

| Table S2 DNA amount and concentration | | | | |  |  |  |  |  |  |
| --- | --- | --- | --- | --- | --- | --- | --- | --- | --- | --- |
|  |  |  |  |  |  |  |  |  |  |  |
| **Library type** | **Individual** | **DNA amount (ng)** | **DNA conc.^1^ (ng/µl)** | **Volume (µl)** | **PCR cycles** | **Library conc.^1^ (nM)** | **Library volume (µl)** | **Average library fragment size (bp)** | **Library yield (ng)** |  |
|  |  |  |  |  |  |  |  |  |  |  |
| **EM-seq** | **1** | 1.0 | 0.5 | 2.0 | 12 | 52.9 | 25.0 | 638 | 547.6 |  |
|  | **1** | 2.0 | 0.5 | 4.0 | 10 | 32.0 | 25.0 | 645 | 334.9 |  |
|  | **1** | 5.0 | 0.5 | 10.0 | 10 | 86.4 | 25.0 | 650 | 911.2 |  |
|  | **1** | 10.0 | 0.5 | 20.0 | 10 | 182.3 | 25.0 | 621 | 1840.0 |  |
|  | **2** | 1.0 | 0.5 | 2.0 | 12 | 76.2 | 25.0 | 595 | 735.6 |  |
|  | **2** | 2.0 | 0.5 | 4.0 | 10 | 29.8 | 25.0 | 669 | 323.5 |  |
|  | **2** | 5.0 | 0.5 | 10.0 | 10 | 63.3 | 25.0 | 640 | 657.3 |  |
|  | **2** | 10.0 | 0.5 | 20.0 | 10 | 136.5 | 25.0 | 619 | 1370.0 |  |
| **PBAT** | **1** | 1.0 | 0.5 | 2.0 | 12 | 33.9 | 50.0 | 528 | 580.8 |  |
|  | **1** | 2.0 | 0.5 | 4.0 | 10 | 27.6 | 50.0 | 543 | 486.3 |  |
|  | **1** | 5.0 | 0.5 | 10.0 | 10 | 33.7 | 50.0 | 561 | 613.5 |  |
|  | **1** | 10.0 | 0.5 | 20.0 | 10 | 185.3 | 50.0 | 556 | 3340.0 |  |
|  | **2** | 1.0 | 0.5 | 2.0 | 12 | 7.9 | 50.0 | 518 | 132.4 |  |
|  | **2** | 2.0 | 0.5 | 4.0 | 10 | 5.6 | 50.0 | 520 | 94.5 |  |
|  | **2** | 5.0 | 0.5 | 10.0 | 10 | 9.7 | 50.0 | 538 | 169.3 |  |
|  | **2** | 10.0 | 0.5 | 20.0 | 10 | 23.1 | 50.0 | 568 | 425.8 |  |
| 1. conc. for concentration | | |  |  |  |  |  |  |  |  |

| Table S3 Conversion efficiency | | | |  |  |  |  |  |  |
| --- | --- | --- | --- | --- | --- | --- | --- | --- | --- |
| Method | Individual | Input (ng) | CHG methylation | CHH methylation | Lambda methylation | pUC19 CpG methylation | Efficiency estimated from CHG^1^ | Efficiency estimated from CHH^2^ |  |
|  |  |  |  |  |  |  |  |  |  |
| EM-seq | 1 | 1 | 2.70% | 2.90% | 2.88% | 94.87% | 97.3% | 97.1% |  |
|  |  | 2 | 0.20% | 0.20% | 0.22% | 96.11% | 99.8% | 99.8% |  |
|  |  | 5 | 0.10% | 0.10% | 0.08% | 95.56% | 99.9% | 99.9% |  |
|  |  | 10 | 0.20% | 0.10% | 0.12% | 96.72% | 99.8% | 99.9% |  |
|  | 2 | 1 | 0.10% | 0.10% | 0.10% | 96.00% | 99.9% | 99.9% |  |
|  |  | 2 | 0.10% | 0.10% | 0.10% | 96.44% | 99.9% | 99.9% |  |
|  |  | 5 | 0.10% | 0.10% | 0.09% | 97.36% | 99.9% | 99.9% |  |
|  |  | 10 | 0.10% | 0.10% | 0.07% | 98.01% | 99.9% | 99.9% |  |
| PBAT | 1 | 1 | 1.1% | 1.0% |  |  | 98.9% | 99.0% |  |
|  |  | 2 | 1.1% | 1.0% |  |  | 98.9% | 99.0% |  |
|  |  | 5 | 1.1% | 1.0% |  |  | 98.9% | 99.0% |  |
|  |  | 10 | 1.1% | 1.1% |  |  | 98.9% | 98.9% |  |
|  | 2 | 1 | 1.3% | 1.3% |  |  | 98.7% | 98.7% |  |
|  |  | 2 | 1.3% | 1.2% |  |  | 98.7% | 98.8% |  |
|  |  | 5 | 1.2% | 1.2% |  |  | 98.8% | 98.8% |  |
|  |  | 10 | 1.3% | 1.2% |  |  | 98.7% | 98.8% |  |
| 1. 100%-CHG methylation | | |  |  |  |  |  |  |  |
| 2. 100%-CHH methylation | | |  |  |  |  |  |  |  |

| Table S4. Raw, trimmed and mapped read number | | | | |  |  |  |  |  |  |  |  |
| --- | --- | --- | --- | --- | --- | --- | --- | --- | --- | --- | --- | --- |
| Library type | Individual | Input (ng) | Raw reads | Clean reads^1^ | Aligned reads | Alignment rate | Unaligned reads | Ambiguously aligned reads | Duplicate reads (removed) | Duplicate reads/clean reads | Unique reads (remaining) | Unique reads/clean reads |
| EMSeq | 1 | 1 | 127902810 | 127873680 | 84187269 | 65.84% | 40030539 | 3655818 | 19093793 | 15% | 65093476 | 50.90% |
|  |  | 2 | 110177132 | 110132408 | 70219861 | 63.76% | 36863599 | 3048897 | 11741864 | 11% | 58477997 | 53.10% |
|  |  | 5 | 103720144 | 103693962 | 65132649 | 62.81% | 35695924 | 2865346 | 10448448 | 10% | 54684201 | 52.74% |
|  |  | 10 | 98342580 | 98308505 | 66420068 | 67.56% | 28858671 | 3029715 | 9903902 | 10% | 56516166 | 57.49% |
|  | 2 | 1 | 101565301 | 101539320 | 71199452 | 70.12% | 27016140 | 3323680 | 15074611 | 15% | 56124841 | 55.27% |
|  |  | 2 | 147771606 | 147722565 | 90063185 | 60.97% | 53607397 | 4051890 | 19564381 | 13% | 70498804 | 47.72% |
|  |  | 5 | 115784479 | 115759275 | 77961466 | 67.35% | 34255832 | 3541922 | 12434423 | 11% | 65527043 | 56.61% |
|  |  | 10 | 88445825 | 88397828 | 59814320 | 67.66% | 25833477 | 2749982 | 7844943 | 9% | 51969377 | 58.79% |
| PBAT | 1 | 1 | 87137209 | 85620218 | 37982801 | 44.36% | 45005077 | 2632326 | 7674871 | 9% | 30307930 | 35.40% |
|  |  | 2 | 141082063 | 138743439 | 60348198 | 43.50% | 74278489 | 4116730 | 11911691 | 9% | 48436507 | 34.91% |
|  |  | 5 | 129371845 | 127255838 | 52212822 | 41.03% | 71408184 | 3634821 | 9595769 | 8% | 42617053 | 33.49% |
|  |  | 10 | 101899010 | 100218737 | 39704414 | 39.62% | 57609725 | 2904576 | 6492008 | 6% | 33212406 | 33.14% |
|  | 2 | 1 | 136095731 | 134197987 | 61559573 | 45.87% | 69031254 | 3607141 | 31942404 | 24% | 29617169 | 22.07% |
|  |  | 2 | 147805755 | 145916383 | 66613929 | 45.65% | 75309506 | 3992919 | 23518982 | 16% | 43094947 | 29.53% |
|  |  | 5 | 65872303 | 65010658 | 28033827 | 43.12% | 35314637 | 1662183 | 5012428 | 8% | 23021399 | 35.41% |
|  |  | 10 | 95653370 | 94229154 | 38500928 | 40.86% | 53437140 | 2291064 | 7054985 | 7% | 31445943 | 33.37% |
| 1. Clean reads: reads after adapter/quality trimming | | | | |  |  |  |  |  |  |  |  |
